# Corticospinal excitability during timed interception depends on the speed of the moving target

**DOI:** 10.1152/jn.00153.2025

**Published:** 2025-07-14

**Authors:** Justin R. McCurdy, Daniel Zlatopolsky, Ria Doshi, Jing Xu, Deborah A. Barany

**Affiliations:** 1 Department of Kinesiology, University of Georgia, Athens, Georgia, United States; 2 Neuroscience Program, University of Georgia, Athens, Georgia, United States; 3 Department of Interdisciplinary Biomedical Sciences, University of Georgia, Athens, Georgia, United States; 4 Regenerative Bioscience Center, University of Georgia, Athens, Georgia, United States

**Keywords:** action preparation, interception, primary motor cortex, target speed, transcranial magnetic stimulation

## Abstract

Successfully intercepting a moving object requires precisely timing the optimal moment to act by integrating information about the target’s visual motion properties. Neurophysiological evidence indicates that activity in the primary motor cortex (M1) during interception preparation is sensitive to both the target’s kinematic features and motor planning. However, how visual motion signals modulate M1 during timed interception remains unclear. In the present study, we applied single-pulse transcranial magnetic stimulation (TMS) over M1 to examine how a target’s kinematics influence corticospinal excitability during interception preparation. Participants were instructed to abduct their right index finger to intercept a target moving horizontally at a constant speed toward a fixed interception zone. Target speed (Fast or Slow) and travel distance (Far or Close) were manipulated while controlling motion duration across conditions. Motor-evoked potentials (MEPs) were elicited at five latencies before target arrival at the interception zone. Consistent with previous behavioral findings, movement initiation occurred earlier for faster targets and was delayed when TMS was applied closer to the target’s arrival. Though MEPs were generally suppressed relative to baseline at earlier time points and facilitated closer to movement initiation, we observed that target speed—but not distance—influenced the time course of MEP modulation. When adjusting for movement initiation times, there was an overall reduced suppression and increased facilitation for faster-moving targets, possibly reflecting a heightened urgency to move. These results suggest M1 activity during interception preparation reflects internal estimates of target motion, which may serve to optimize interception timing and performance.

## NEW & NOTEWORTHY

When intercepting a moving object, like catching a ball, we need to continuously combine visual motion signals to predict the object’s future location and enable accurate movement. Here, we show that preparatory suppression and facilitation of corticospinal excitability depend on the speed, but not the distance, of the moving target. These findings reveal that differences in interception timing are closely linked to changes in motor system excitability.

## INTRODUCTION

Intercepting moving objects with the hand, like catching a ball or stopping a water bottle from rolling off the table, relies on integrating a continuous prediction of the object’s future location with estimates of the current hand position (1-4). Humans achieve high spatial and temporal precision during interceptive movements in part by estimating time-to-contact (TTC), defined as the time remaining until a moving object or intercepting effector reaches a specific location (1-5). When intercepting objects moving at constant speed, humans use both kinematic (e.g., speed and distance) and timing cues to accurately estimate TTC (6). Under full vision conditions, subjects initiate fast interceptive movements earlier and/or move faster when target speeds are increased (7, 8), implying that accurate integration of kinematic information is crucial for preparing interception movements (4, 9-11).

Relative to the extensive literature on reaching to static targets (12, 13), the neural substrates responsible for estimating TTC to guide interceptive timing remain largely underexplored (14). In addition to engaging visuomotor fronto-parietal networks (15-17), estimating TTC while preparing to intercept a moving target is associated with neural activity in the middle temporal area (MT/V5 +), subcortical regions, and the cerebellum (18-23). Most of these signals are expected to converge within primary motor cortex (M1) to specify and release descending motor commands (24). Evidence from single-neuron recordings in nonhuman primates and from human neuroimaging indicates that the modulation of M1 activity reflects both time-varying aspects of motor planning and the processing of visual information during estimation of TTC (3, 18, 25-27).

In humans, single-pulse transcranial magnetic stimulation (TMS) has been extensively used to study corticospinal excitability (CSE) modulation during action preparation (reviewed in Refs. 28 and 29). In delayed reaction time (RT) and predictive timing tasks, in which a cue specifying an upcoming movement is followed by an external or internal imperative signal to move, a consistent pattern of CSE modulation before movement onset is observed. Typically, CSE is suppressed relative to baseline, followed by rapid facilitation in the agonist muscle, coupled with increasing suppression in the antagonist muscle (30-40). In addition, the TMS pulse itself affects timing of the motor response; in simple RT tasks, TMS applied before onset can shorten RT, whereas in predictive timing tasks, TMS delays movement onsets (30, 41-43). Though the functional significance of these TMS-elicited changes in CSE and movement is unresolved, it is thought to reflect a transitional state that primes the motor system for voluntary movement, involving preparatory changes in CSE that ready muscles to initiate the upcoming action (30, 44, 45).

Although previous studies have explored CSE during anticipatory actions in response to moving stimuli (30, 46, 47), it is unclear how systematically varying kinematic motion properties may affect CSE during motor preparation for interception. In this study, we investigated how two TTC variables—target speed and distance—affect CSE when preparing to intercept moving targets with the hand. We measured TMS-elicited motor-evoked potentials (MEPs) in the task-relevant right first dorsal interosseous (FDI) and task-irrelevant abductor digiti minimi (ADM) muscles as participants prepared to abduct their index finger to coincide with a moving target’s arrival at a fixed interception location. On each trial, we varied the target’s speed (Fast or Slow) and starting distance (Close or Far), along with the timing of the TMS pulse relative to the ideal interception time. We hypothesized that faster targets and closer starting distances would lead to increased modulation of excitability due to heightened response urgency. In addition, we hypothesized that if M1 receives information about target motion kinematics to estimate TTC, then the time course of CSE suppression and facilitation would align with interception onset.

## MATERIALS AND METHODS

### Participants

We recruited 23 healthy, right-handed participants (12 M, 21.8 ± 3.6 yr) with no known history of neurological disorders, no contraindications to TMS, and normal or corrected-to-normal vision to complete the experiment. All participants were naïve to the purpose of the study, provided written informed consent, and were compensated for their time. One participant was excluded for poor behavioral performance (see Data Analysis), resulting in a final sample of 22 participants (11 M, mean age 21.9 ± 3.7 yr). The experimental procedures were approved by the Institutional Review Board at the University of Georgia.

### Experimental Setup

Participants were seated in front of the task monitor (1,920 × 1,080 resolution, 60-Hz refresh rate) at a desk with their right hand resting on a digitizing trackpad (WACOM Intuous Pro Large). The experiment and stimulus presentation were controlled using PsychoPy 3 Version 2022.2.4 (48). The trackpad was positioned so that participants could rest both arms comfortably on the desk while placing their chin in a custom stand ~58 cm from the center of the screen. Participants wore earplugs for the duration of the study and a sleeve over their index finger to enhance trackpad sensitivity. Surface electromyography (EMG) electrodes were attached to the FDI and ADM muscles of the participant’s right hand. EMG activity was recorded using a Delsys Bagnoli desktop system (Delsys Inc., Boston, MA). EMG signals were amplified 1,000 times, bandpass filtered (20–450 Hz), and sampled at 5,000 Hz through a data acquisition board (Micro 1401-4, Cambridge Electronic Design, Cambridge, UK) and CED Signal software.

### Transcranial Magnetic Stimulation

TMS was applied through a 70-mm, figure-of-eight coil driven by a DuoMAG MP magnetic stimulator (Deymed Diagnostics sro, Hronov, Czech Republic). MEPs were elicited from the right FDI by applying TMS over the hand area of the left M1. The coil was placed tangentially on the scalp with the handle oriented toward the occiput and laterally from the midline at a 45° angle. Before the experimental session, the FDI motor hotspot (i.e., the optimal location for eliciting MEPs in the target muscle) was identified on each participant by eliciting TMS every 4 s and systematically repositioning the coil. At the beginning of each experimental session, a model of the brain was scaled to the participant’s cranial dimensions using a neuronavigational tracking system (BrainSight, Rogue Research, Montreal, Canada) to optimize and replicate coil position throughout the session. Resting motor threshold (rMT) was defined as the minimal TMS intensity required to evoke peak-to-peak MEP amplitudes of ~50 μV in the target muscle for 5 of 10 consecutive trials (49). The average rMT for this experiment was 43.3% (±4.5) of the maximum stimulator output. Stimulator intensity during the task blocks was set to 115% of the participant’s rMT. TMS timings were controlled by sending TTL signals from the task computer to the stimulator through CED Signal.

### Interception Task

**Figure 1.**
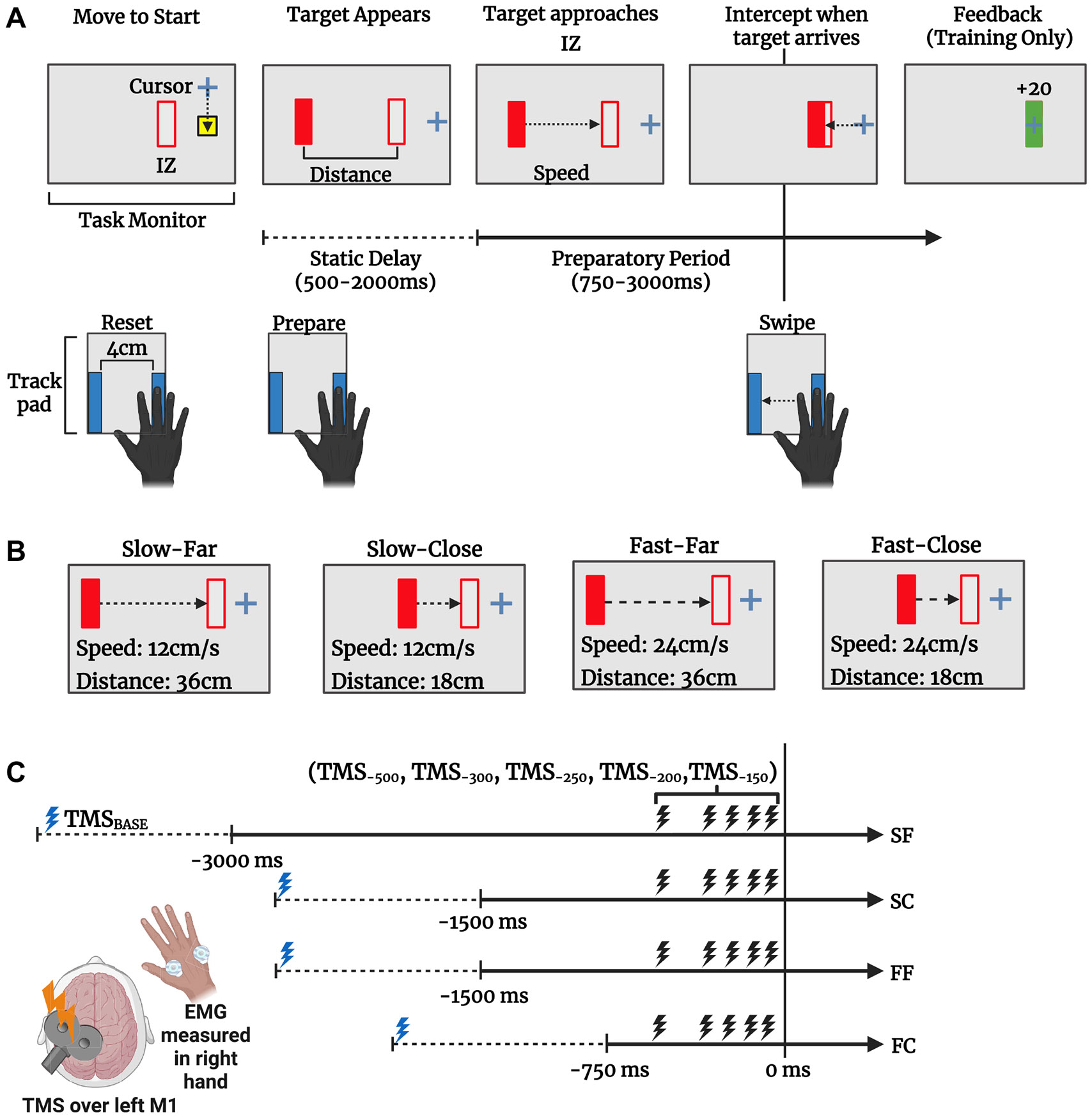
Interception task design. *A*: participants (*n* = 22) prepared to swipe their right index finger on a trackpad to intercept a moving target to coincide with the target time of arrival at a predefined interception zone (IZ) on the screen. During training, they were provided feedback about the movement timing. *B*: during each block, the four trial conditions (Slow-Far, Slow-Close, Fast-Far, and Fast-Close) were presented in randomized order, so participants needed to judge target motion to time their movements. Note that the four combinations of target speed and distance resulted in three unique preparation period durations (3,000 ms for Slow-Far, 1,500 ms for Slow-Close and Fast-Far, and 750 ms for Fast-Close). *C*: TMS was applied over the left M1 representation of the first dorsal interosseous (FDI) muscle at target presentation (baseline) or at 1 of 5 time points (500, −300, −250, −200, or −150 ms) relative to target-IZ overlap during the preparation period. EMG was measured in both the task-relevant FDI muscle and the task-irrelevant abductor digit minimi (ADM) muscle. TMS, transcranial magnetic stimulation. Figure created with a licensed version of BioRender.com.

Participants performed an interception task in which the goal was to move an on-screen cursor representing their right index finger position into a predefined interception zone (IZ) timed to match the arrival of a moving target (Fig. 1A). At the start of each trial, participants were instructed to move an on-screen cursor (black cross, 1 × 1 cm) into a fixed start position on the trackpad and remain there until the target appeared. Once the cursor was in the start position for 500 ms, a red rectangular target (0.65 × 4 cm) would appear on the screen at a fixed distance to the left of the IZ (0.65 × 4 cm). After a variable delay (500–2,000 ms, uniform distribution), the target would begin approaching the interception zone at a constant horizontal speed. Participants were instructed to make a quick swiping movement by abducting their right index finger to intercept the target as it arrived at the IZ. During practice, participants were reminded to only move their index finger while keeping all other fingers at rest.

On each trial, the target started at either 36 cm (Far) or 18 cm (Close) from the IZ and approached the IZ at either 12 cm/s (Slow) or 24 cm/s (Fast). Figure 1B depicts the four unique trial conditions based on the target’s visual motion properties: Fast-Close, Fast-Far, Slow-Close, and Slow-Far. Target motion durations were 750 ms for Fast-Close, 3,000 ms for Slow-Far, and matched at 1,500 ms for the Fast-Far and Slow-Close conditions to test for the effect of target kinematics independent of preparation time (39). After 20 familiarization trials, participants performed two training blocks of 52 trials in which no TMS was applied. During the training blocks, participants received three forms of feedback about the distance of the target from IZ at the time of interception to help improve response accuracy. First, the target stopped moving at the time of interception and briefly (200 ms) remained in a static position before disappearing. Second, a rewarding “coin” sound played if the target was intercepted within the IZ. Finally, a score (+20, +10, +5) was shown after each trial based on their spatial accuracy, with a cumulative score summary at the end of each training block.

After both training blocks were complete, participants performed six task blocks of 48 trials each. During task blocks, both the trial condition and time of TMS delivery were pseudorandomized based on the block order assigned to the participant, and no performance feedback was provided. During 44 of the 48 trials in each task block, TMS was applied at either the start of the trial to measure within-task baseline (TMS_BASE_; *n* = 4 per block) or at one of five time points relative to the target’s arrival (i.e., 500, 300, 250, 200, or 150 ms before target-IZ overlap; *n* = 8 each per block). This resulted in a total of 24 in-task baseline TMS trials and 12 TMS trials per unique trial condition by time point combination. In the remaining four of the 48 trials in each task block (24 total trials), no TMS was applied to minimize expectancy effects (50).

### Data Analysis

One participant was excluded from data analysis for low interception accuracy (<50% of targets intercepted within IZ). Dependent variables were extracted from the EMG recordings and the finger position data on the trackpad. All EMG recordings were analyzed offline using the MATLAB VETA toolbox (51). Interception performance was quantified by movement onset, movement time, and spatial accuracy. Movement onset was identified as the first time point at which the EMG activity in the FDI muscle exceeded three standard deviations of the mean of the rectified signal for the entire trial epoch and was >0.5 mV, relative to the target’s arrival time. Movement time was defined as the duration from EMG-derived movement onset to the time at which the IZ was reached. Spatial error was defined as the absolute value of the relative distance between the target and IZ at the time the finger position entered the IZ. Timing error was defined as the difference between the time the target and finger position entered the IZ (negative timing error corresponds to earlier interceptions).

CSE was indexed by the TMS-elicited MEP peak-to-peak amplitude. The root mean square (RMS) value of the background EMG activity was calculated for the 300 ms before the TMS artifact. Trials were rejected from analysis when the background RMS was ±2 standard deviations from the participant’s average RMS (1.7% of all trials). Trials in which EMG activity was observed within 300 ms of the MEP and trials in which the EMG burst onset preceded the TMS pulse were excluded from analysis (0.8% of all trials). These steps were performed to prevent the contamination of the MEP measurements from fluctuations in background EMG activity (52). Lastly, trials in which the EMG burst onset was detected more than 300 ms before or after the target’s TTC were removed to account for behavioral outliers (0.7% of all trials). Altogether, a total of 165 trials (3.1% of total trials) across all participants were excluded from this dataset. The peak-to-peak amplitude of the EMG signal was calculated during the 20–50 ms window after TMS (i.e., the normal range at which an MEP is expected to occur). Each participant’s MEP values were normalized to the participant’s average within-task baseline MEP amplitude.

### Statistical Analysis

Interception performance (i.e., movement onset, movement time, spatial error, and timing error) in blocks with and without TMS were assessed using a linear mixed-effects model (LMM), with target speed (Fast or Slow) and starting distance (Close or Far) as fixed effects and participant as a random effect.

Normalized MEP peak-to-peak amplitudes were first analyzed with trials collapsed across target speed and distance conditions for the task-relevant FDI muscle and task-irrelevant ADM muscle using an LMM with TMS time point as a fixed effect and participant as a random effect. Next, we assessed MEP amplitudes for the FDI and ADM muscles separately, with target speed (Fast vs. Slow), target distance (Close vs. Far), and TMS time point as fixed effects, and participant as a random effect. For all MEP analyses, the root mean square (RMS) of pre-MEP background EMG activity over the 300 ms window preceding the TMS artifact was included as a continuous covariate.

To further inspect the CSE results for the task-relevant FDI muscle, we performed a growth curve analysis (53), averaging over the levels of target speed. The overall time course of MEPs was modeled with a second-order (quadratic) orthogonal polynomial, including fixed effects of target speed (Fast vs. Slow; within-participants) on all time terms. The model also included participant random effects on all time terms, participant-by-condition random effects on all time terms, and the RMS of the background EMG as a continuous covariate. Parameter estimate degrees of freedom and corresponding *P* values were estimated using Satterthwaite’s method (54).

Type III ANOVAs were conducted on each nested LMM to determine significant main effects and interactions, with the α level set to 0.05. Post hoc comparisons were conducted on the estimated marginal means using the Holm correction (55).

## RESULTS

### Earlier Movement Onsets for Faster Target Speeds

**Figure 2.**
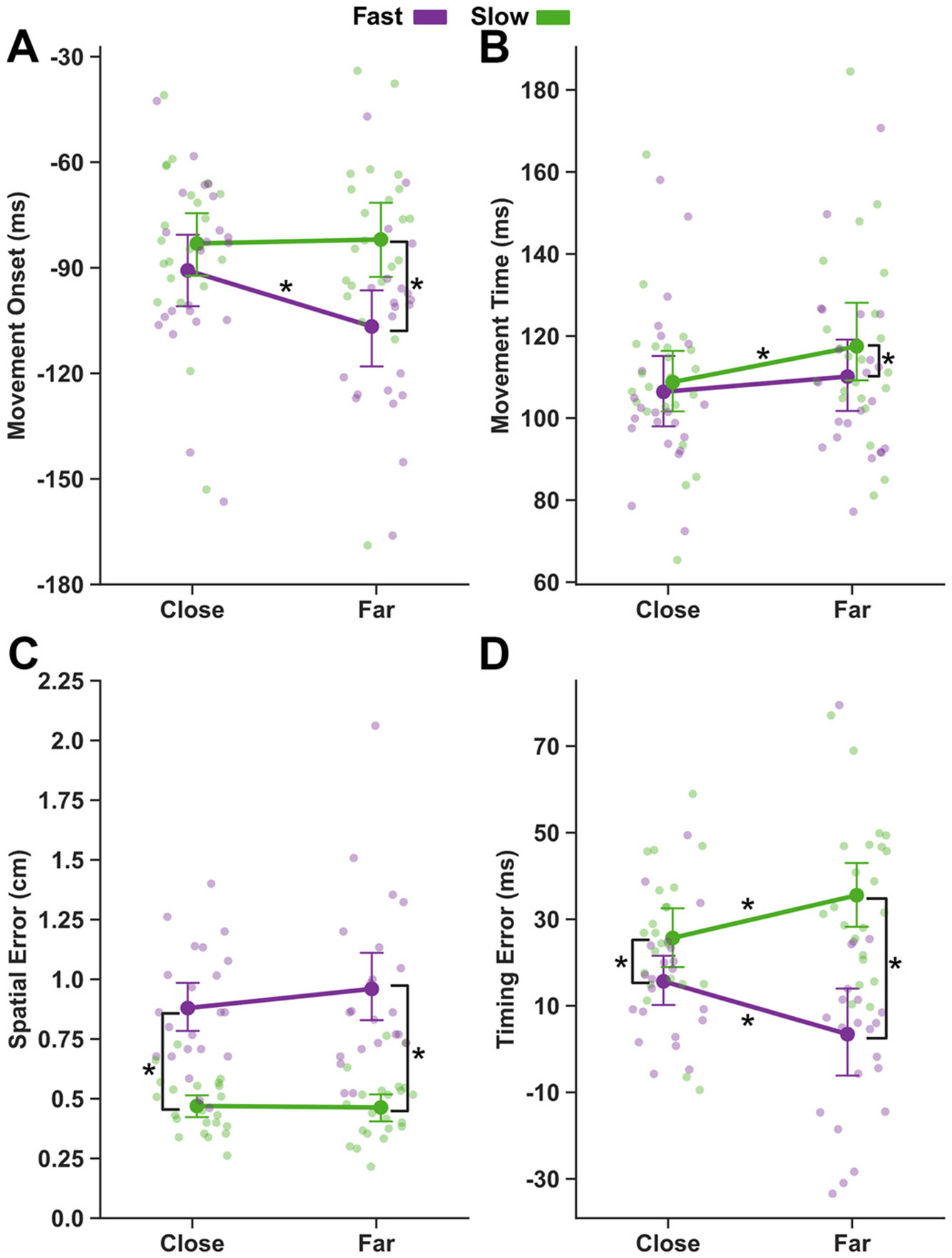
Target speed and preparation duration affected interception characteristics. *A*: movement onset (ms) was earlier for fast targets at far-starting distances. *B*: movement time (ms) was significantly faster for Fast-Close (750 ms duration) than Slow-Far (3,000 ms duration). *C*: spatial error (cm) was larger in fast target conditions. *D*: timing error (ms) was larger for slow targets. Each dot corresponds to a participant’s (*n* = 22) mean in a given condition. Error bars correspond to the 95% confidence interval of the group-mean estimate. *P < 0.05 between conditions (Holm-corrected). Figure created with a licensed version of BioRender.com.

To assess performance differences driven by the target’s motion properties independent of stimulation, behavioral data were first evaluated from the last block of trials without TMS (Fig. 2). Across all trial conditions, movements were initiated on average 91.7 ms (± 24.4 ms) before the target’s arrival (Fig. 2A). There was a significant main effect of target speed (*F* = 27.53, *P* < 0.001), indicating that participants initiated movements earlier for faster-moving targets. The main effect of starting distance was not significant (*F* = 3.54, *P* = 0.073). There was significant interaction between target speed and starting distance (*F* = 7.77, *P* = 0.005), suggesting that movement onsets were earlier for faster-moving targets only at farther starting distances. Post hoc comparisons confirmed significant differences between Fast-Close and Fast-Far (*t* = 3.26, *P* = 0.008), Slow-Close and Fast-Far (*t* = 4.84, *P* < 0.001), and Fast-Far compared with Slow-Far (*t* = −5.63, *P* < 0.001), indicating that faster targets induced earlier movement onsets even with the same motion duration.

Movement times (Fig. 2B) were significantly faster when the target started from the close position (*F* = 9.70, *P* = 0.002) and when approaching at higher speed (*F* = 6.12, *P* = 0.022). There was no significant interaction between target speed and distance (*F* = 1.71, *P* = 0.191). Post hoc tests showed movement times were significantly longer in the Slow-Far condition (all *P*’s < 0.05), with no significant differences between any other condition pairs (all *P*’s > 0.05). In addition, grouping the movement times based on motion duration (Fast-Close; 750 ms, Fast-Far and Slow-Close; 1,500 ms, Slow-Far; 3,000 ms) revealed a significant main effect (*F* = 8.56, *P* < 0.001). Post hoc tests showed that movement times were significantly slower at 3,000 ms than at 750 ms (*t* = −3.96, *P* < 0.001) or 1,500 ms (*t* = −3.32, *P* = 0.002), with no difference between 750 and 1,500 ms (*t* = −1.26, *P* = 0.210). This suggests that movement times were likely influenced mainly by the duration of the preparatory period.

Participants intercepted 76% (± 8.7) of targets within the IZ, indicating overall accurate task performance. To more closely examine interception accuracy, we calculated spatial and timing errors on each trial (Fig. 2, C and D). Spatial errors were larger for fast than for slow targets (*F* = 111.62, *P* < 0.001), but did not differ across levels of start distance (*F* = 1.00, *P* = 0.329). The interaction effect was also not significant (*F* = 1.73, *P* = 0.189). Timing errors were smaller for fast targets (*F* = 51.25, *P* < 0.001), with no main effect of start distance (*F* = 0.07, *P* = 0.796). There was also a significant interaction between target speed and start distance (*F* = 15.98, *P* < 0.001), indicating that faster targets led to smaller timing errors, particularly when the target started from a further distance. Post hoc tests revealed significant differences between each of the four conditions (all *P*’s < 0.05). Differences in timing error were likely driven by differences in movement onset (compare Figs. 2A and 2D). Together, these results indicate that target speed influenced interception performance: intercepting fast-moving targets required more temporal precision to intercept the target within the IZ, leading to lower timing error but increased spatial error.

### TMS Effects on Interception Performance Varied with Target Speed and Distance

**Figure 3.**
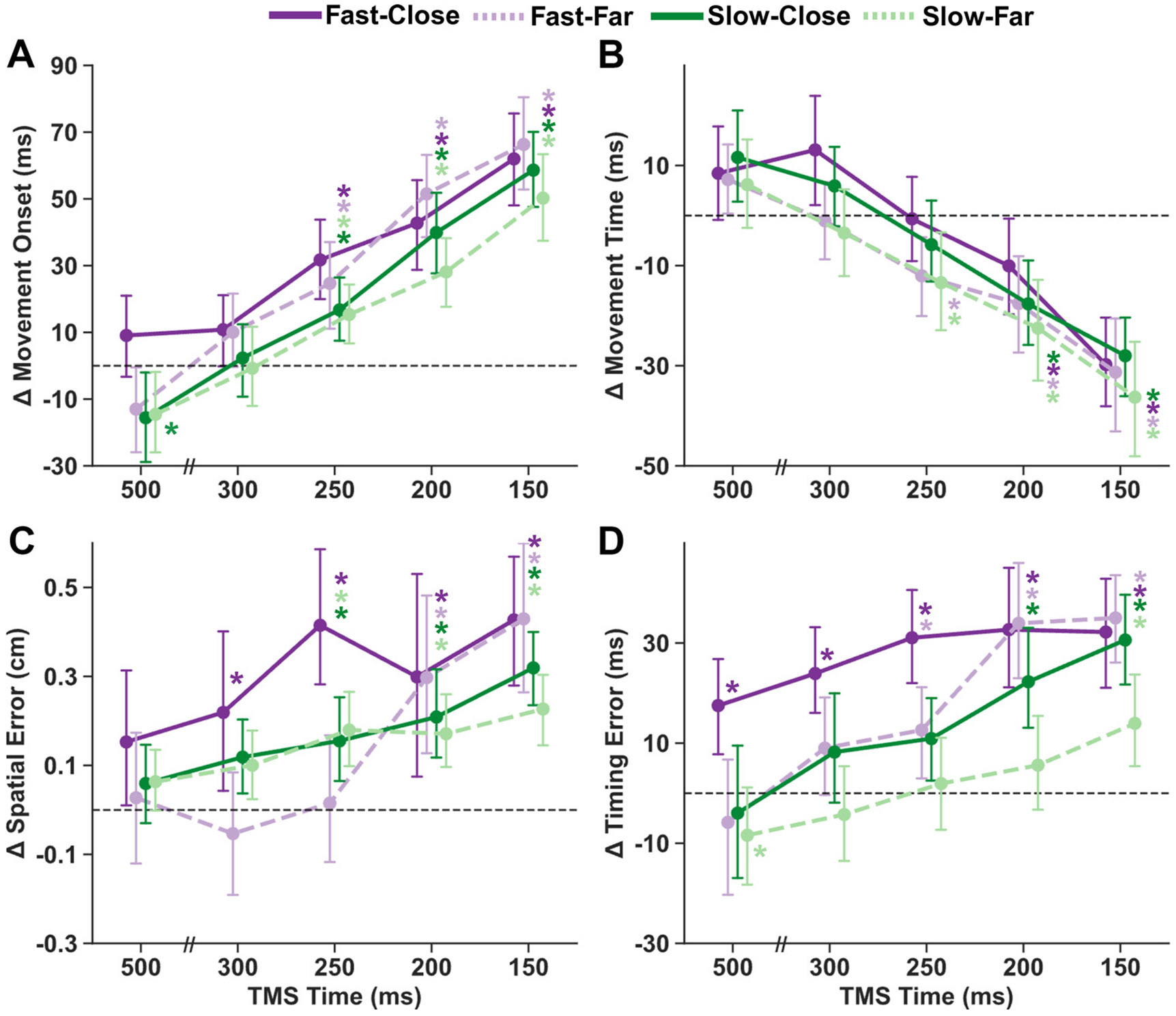
TMS applied during movement preparation disrupts interception timing. All plots show average change relative to the participant’s average on trials without TMS. Purple (green) lines represent fast (slow) target trials, and solid (dotted) lines represent close (far) start distance trials. *A*: movement onset was delayed more at TMS time points closer to the target arrival and for fast target conditions. *B*: movement time sped up at TMS time points closer to target arrival and for far target conditions. *C*: spatial error tended to increase during TMS trials, especially at later time points. *D*: timing error increased at all time points for fast-close and increased at later time points for the other conditions. Circles show the group mean (*n* = 22) for each condition at each time of TMS delivery. All error bars correspond to the 95% confidence interval of the group-mean estimate. *P < 0.05 different from zero (Holm-corrected). TMS, transcranial magnetic stimulation. Figure created with a licensed version of BioRender.com.

To examine the influence of applying TMS on interception performance, the average of each subject’s movement onset, movement time, spatial error, and timing error were calculated for each combination of target condition (Fast-Close, Fast-Far, Slow-Close, and Slow-Far) and TMS delivery time (500, 300, 250, 200, and 150 ms before target’s arrival) and then normalized to each subject’s average during the second block of trials without TMS (Fig. 3).

For change in movement onset (Fig. 3A), there was a significant main effect of time of TMS delivery (*F* = 80.60, *P* < 0.001). In general, TMS delayed movement onset more when applied closer to the target’s time of arrival (all *P*’s < 0.05), suggesting that TMS during late stages of movement preparation interfered with response execution. There was a significant main effect of target speed (*F* = 13.51, *P* = 0.001), but not of start distance (*F* = 1.91, *P* = 0.182), indicating that the change in movement onset was greater for faster-moving targets. There was a significant three-way interaction between target speed, distance, and time of TMS delivery (*F* = 9.73, *P* < 0.001). Post hoc tests revealed that at the earliest time point (TMS_500_), TMS sped up movement onsets for the Slow-Close (*t* = −3.81, *P* < 0.001) condition, but not for the Fast-Far (*t* = −2.13, *P* = 0.072), Slow-Far (*t* = −2.26, *P* = 0.072) or Fast-Close (*t* = 1.40, *P* = 0.163) conditions. In contrast, movement onset was significantly delayed for each condition at TMS_250_, TMS_200_, and TMS_150_ (all *P*’s < 0.05). Together, these results suggest that TMS delays movement initiation more when applied closer to movement initiation and for faster-moving targets.

Analysis of movement times suggests that participants sped up or slowed down their movements to partially compensate for differences in movement onset (Fig. 3B). There was a significant main effect of TMS stimulation time (*F* = 38.25, *P* < 0.001)—movement times were shorter as TMS was delivered closer to target arrival time relative to blocks with no TMS. There was a significant main effect of target distance (*F* = 12.00, *P* = 0.002) and speed (*F* = 5.00, *P* = 0.037), indicating that TMS shortened movement times more for Far and Slow target conditions. Relative to their average on blocks with no TMS, movement times were not significantly different at TMS_500_ and TMS_300_ for all conditions (all *P*’s > 0.05). At TMS_250_, movement times were significantly decreased for Fast-Far (*t* = −2.56, *P* = 0.031) and Slow-Far (*t* = −2.77, *P* = 0.022) and not significantly different from zero for Fast-Close (*t* = −0.11, *P* = 0.917) and Slow-Close (*t* = −1.30, *P* = 0.390). At both the latest TMS delivery time points (TMS_200_ and TMS_150_), movement times were significantly shortened for all conditions (all *P*’s < 0.05). Altogether, TMS during late stages of movement preparation resulted in shorter movement times, especially when the target started from farther away.

TMS increased spatial error, especially when delivered closer to the target’s arrival (main effect of TMS time: *F* = 10.98, *P* < 0.001; Fig. 3C). A significant three-way interaction between target speed, distance, and TMS time (*F* = 6.46, *P* < 0.001) revealed this time-dependent increase in spatial error depended on target motion properties: at TMS_250_, spatial error was significantly elevated across all conditions except Fast-Far (*t* = 0.12, *P* = 0.909), whereas at TMS_300_, increased error was observed only for the Fast-Close condition (*t* = 2.87, *P* = 0.016). Notably, no significant effects emerged at the earliest stimulation time (TMS_500_; all *P*’s > 0.05). In addition, there was a significant two-way interaction between target speed and distance (*F* = 15.59, *P* < 0.001), along with a main effect of target distance (*F* = 6.99, *P* = 0.016), but not target speed (*F* = 2.90, *P* = 0.103), indicating increased spatial error for the Close target distances, especially at Fast target speeds.

Interception timing error was also significantly modulated by TMS delivery time (*F* = 24.21, *P* < 0.001), with larger errors observed when TMS was delivered closer to target arrival (Fig. 3D). A significant three-way interaction between target speed, distance, and TMS time (*F* = 6.24, *P* < 0.001) revealed that TMS affected timing differentially across conditions: for the Fast-Close condition, TMS led to increased error relative to No TMS at all stimulation time points (all *P*’s < 0.01), whereas in the Fast-Far, Slow-Close, and Slow-Far conditions, error magnitude progressively increased with later stimulation. There were significant main effects of both speed (*F* = 34.50, *P* < 0.001) and distance (*F* = 12.18, *P* = 0.002), suggesting that TMS disrupted timing more for Fast target speeds and Close target distances.

Collectively, these results suggest that TMS interferes with the late stages of movement preparation to disrupt interception performance, and that the ability to compensate for the effects of TMS is influenced by both target speed and distance.

### Corticospinal Excitability Depended on Target Speed, but not Distance

**Figure 4.**
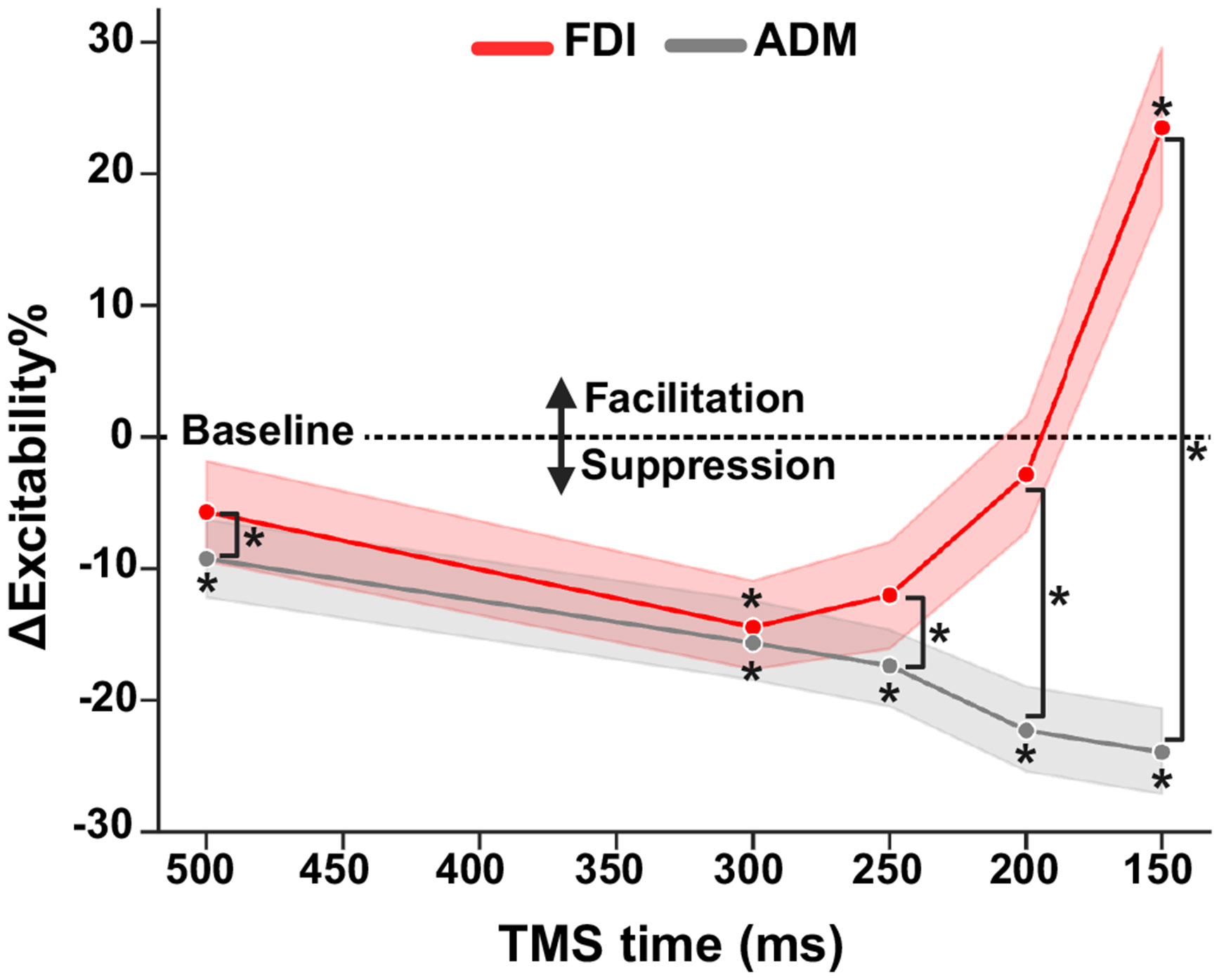
Early task-relevant suppression and late facilitation during interception preparation. Timeline of corticospinal excitability for task-relevant (FDI) and task-irrelevant (ADM) effectors across all target speed and distance conditions, showing early preparatory suppression and late facilitation. Error bars correspond to the 95% confidence interval estimated via trial-level bootstrapping within each participant (*n* = 22). *P < 0.05 different from zero or between FDI and ADM muscles (Holm-corrected). ADM, abductor digit minimi; FDI, first dorsal interosseous. Figure created with a licensed version of BioRender.com.

To evaluate CSE modulation, we calculated changes in MEP amplitudes normalized to within-task baseline at each TMS delivery time for the task-relevant FDI and task-irrelevant ADM muscles. Figure 4 shows the average normalized amplitude of MEPs evoked in FDI and ADM at each TMS time across all conditions. There were significant main effects of TMS time (*F* = 9.04, *P* < 0.001) and muscle (*F* = 29.58, *P* < 0.001), indicating CSE changed over the preparatory period and that overall CSE was significantly higher for FDI relative to ADM. Increased pre-MEP background EMG activity was also associated with higher MEP amplitudes (*F* = 90.07, *P* < 0.001). A significant interaction between TMS time and muscle (*F* = 56.85, *P* < 0.001) revealed that CSE was modulated differently throughout the preparatory period depending on the muscle’s relevance to the upcoming movement. Follow-up tests showed that mean FDI CSE was suppressed relative to baseline at TMS_300_ (*t* = −2.17, *P* = 0.030) and then became significantly facilitated immediately before movement execution at TMS_150_ (*t* = 5.52, *P* < 0.001).

In contrast, excitability of the task-irrelevant muscle (ADM) was significantly suppressed relative to baseline at all TMS time points (all *P*’s < 0.05). Relative to TMS_500_, there was a significant increase in suppression at the later TMS_200_ (*t* = 3.52, *P* = 0.004) and TMS_150_ (*t* = 3.95, *P* < 0.001) time points. Post hoc pairwise tests between the FDI and ADM muscles showed greater suppression of the ADM muscle at all but the TMS_300_ time point (*P*’s < 0.05). Together, these results confirm that during movement preparation, task-relevant muscles show a pattern of CSE suppression followed by facilitation, whereas task-irrelevant muscles undergo a sustained and increasing suppression (30, 56, 57).

**Figure 5.**
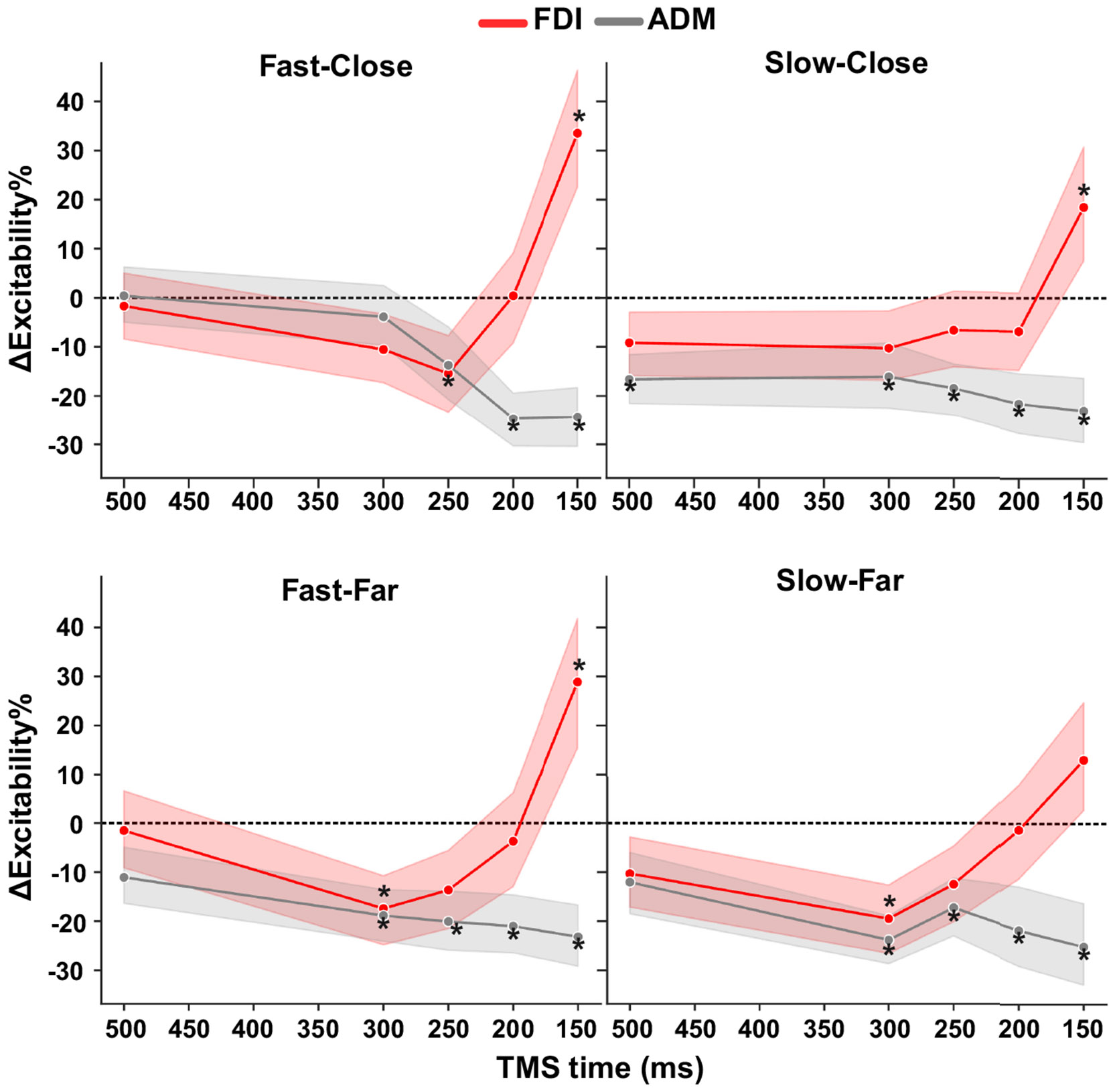
Corticospinal excitability (CSE) during interception preparation shows higher late facilitation for faster-moving targets and earlier suppression for slower-moving targets. Each subplot shows the time course of change in CSE relative to baseline for relevant (FDI) and nonrelevant (ADM) muscles for each target motion condition. Error bars correspond to the 95% confidence interval estimated via trial-level bootstrapping within each participant (*n* = 22). *P < 0.05 from zero (Holm-corrected). ADM, abductor digit minimi; FDI, first dorsal interosseous. Figure created with a licensed version of BioRender.com.

Figure 5 shows the average change in CSE from within-task baseline at each TMS time point for each of the four target motion conditions. To assess MEP amplitudes in the FDI muscle, we used a linear mixed model with the target’s speed (Fast vs. Slow), start distance (Close vs. Far), and time of TMS relative to the target’s time of arrival (−500, −300, −250, −200, and −150 ms) as fixed factors, participant as a random factor, and background activity (RMS of pre-MEP EMG) as a continuous covariate. As before, there was a significant effect of background EMG (*F* = 18.16, *P* < 0.001), confirming larger MEP responses with higher levels of pre-MEP activation. Interestingly, there was a significant interaction between TMS time point and target speed (*F* = 3.71, *P* = 0.005), but not distance (*F* = 0.07, *P* = 0.792), suggesting that CSE was modulated throughout interception preparation differently depending on the speed of the target. In addition, main effects of target speed (*F* = 5.02, *P* = 0.036) and TMS time (*F* = 15.62, *P* < 0.001) were observed, indicating increased excitability for faster-moving targets and at later time points.

Follow-up tests showed that when TMS was delivered at 150 ms before the target’s arrival at IZ, there was a significant increase in the MEP amplitude relative to baseline for the Fast-Close (*t* = 4.53, *P* < 0.001), Fast-Far (*t* = 4.18, *P* < 0.001), and Slow-Close (*t* = 2.62, *P* = 0.02) motion conditions, with a smaller increase in the Slow-Far condition that did not significantly differ from baseline (*t* = 1.76, *P* = 0.078). In contrast, at 200 ms and 250 ms before target TTC, MEP amplitudes for all four trial conditions were not significantly different from zero (all *P*’s > 0.05). MEPs elicited at 300 ms were significantly reduced for the Fast-Far (*t* = −2.47, *P* = 0.040) and Slow-Far (*t* = −2.73, *P* = 0.025) conditions, and not significantly different from baseline for the Fast-Close (*t* = −1.54, *P* = 0.245) and Slow-Close (*t* = −1.54, *P* = 0.245) conditions. Finally, at our earliest TMS time point (500 ms from target-IZ overlap), there was some evidence of MEP suppression for both slow speed conditions (Slow-Far; *t* = −1.54, *P* = 0.497, Slow-Close; *t* = −1.34, *P* = 0.538), though this was not significantly different from baseline.

**Figure 6.**
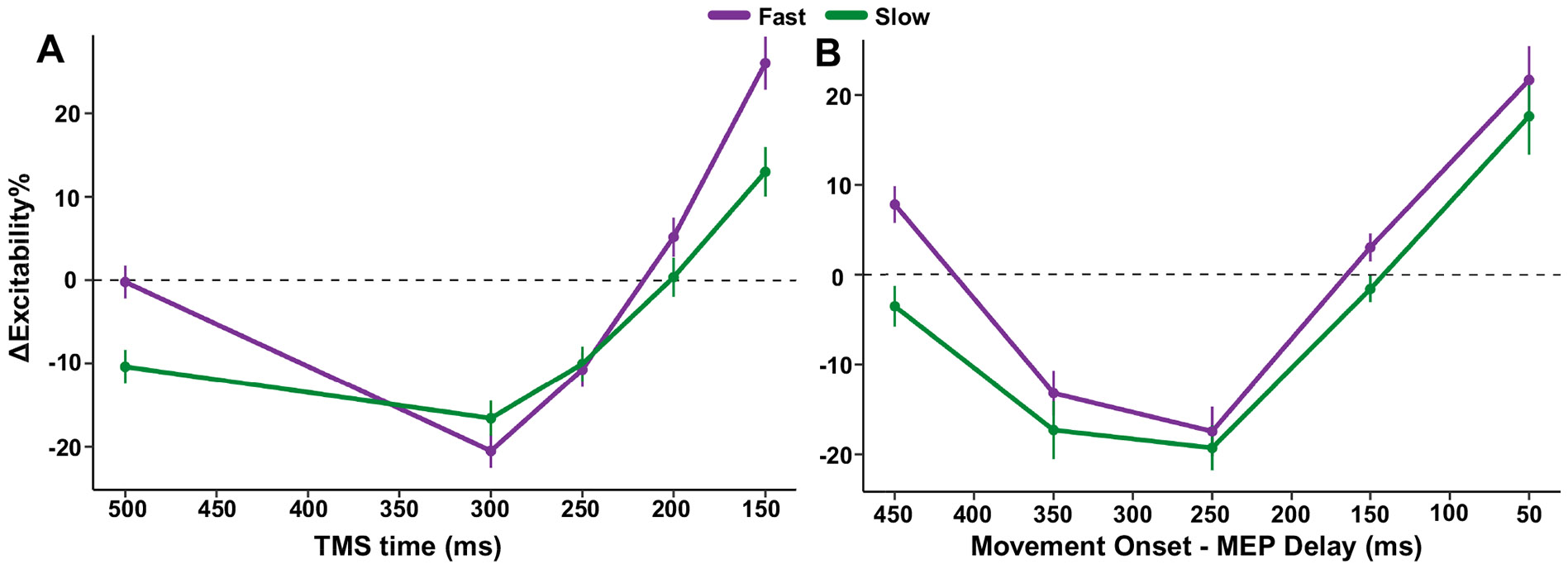
Increased overall excitability for faster-moving targets when adjusting for movement onset. Growth curve analysis for FDI excitability between speed conditions relative to TMS time (*A*) and movement onset (*B*). Error bars correspond to the 95% confidence interval around the model-predicted means. FDI, first dorsal interosseous; TMS, transcranial magnetic stimulation. Figure created with a licensed version of BioRender.com.

To further examine the interaction between target speed and time of TMS delivery for the FDI muscle, we performed a growth curve analysis (53) averaged across the levels of target speed (Fig. 6A; see Statistical Analysis for details). Here, we found a significant effect of target speed on the intercept term, indicating lower overall MEP amplitude proportions for the Slow condition relative to the Fast condition (Estimate = −4.73, SE = 2.04, *P* = 0.03). There was also a significant effect on the quadratic term, indicating shallower curvature in the Slow condition relative to the Fast condition (Estimate = −14.48, SE = 4.98, *P* = 0.007). All other effects of target speed were not significant.

Given that behaviorally, we observed earlier movement onsets for faster-moving targets (see Figs. 3 and 4), we conducted a second growth curve analysis in which we computed the difference between the time of TMS delivery and movement onset so that MEPs would be aligned to the EMG-based time of movement onset instead of the target’s arrival (Fig. 6B). Trials were sorted into 100 ms time bins centered around 450, 350, 250, 150, and 50 ms before movement. There was still a significant effect of target speed on the intercept term, indicating overall higher levels of CSE for fast targets relative to slow targets (Estimate = −6.31, SE = 2.87, *P* = 0.04). However, there was no longer a significant effect of speed on the quadratic term, indicating that the curvature of preparatory suppression was similar between slow and fast trials (Estimate = −10.4, SE = 2.87, *P* = 0.09). All other effects of target speed were not significant. These results suggest that, though faster target speeds heighten CSE overall, the specific pattern of CSE modulation is relative to response execution.

In the task-irrelevant muscle (ADM; gray lines in Fig. 5), there were significant effects of background EMG (*F* = 135.38, *P* < 0.001) and time of TMS (*F* = 7.98, *P* < 0.001), but not for target speed (*F* = 3.35, *P* = 0.081) or distance (*F* = 4.27, *P* = 0.052). In addition, there was a significant two-way interaction between target distance and TMS delivery time (*F* = 2.63, *P* = 0.032) and target speed and TMS delivery time (*F* = 2.64, *P* = 0.032), indicating that ADM showed greater early suppression at farther starting distances and slower target speeds (see Fig. 5). Post hoc tests showed that ADM MEP amplitudes at later time points (TMS_150_, TMS_200_, and TMS_250_) were significantly reduced for all conditions (all *P*’s < 0.01). However, at the earliest time point (TMS_500_), MEPs were only significantly reduced for the Slow-Close condition (*t* = −3.09, *P* = 0.008), and at 300 ms before the target’s arrival, amplitudes were significantly reduced for the Slow-Close, Slow-Far, and Fast-Far (all *P*’s < 0.01) conditions, but not for Fast-Close (*t* = −1.00, *P* = −0.315) conditions. These results suggest that the early suppression of task-irrelevant muscles is apparent only with longer (>750 ms) preparatory periods.

## DISCUSSION

In the present study, we examined how target speed and distance influence TMS-evoked motor excitability during the preparation phase of manual interceptions. We hypothesized that if M1 receives kinematic information regarding TTC, then CSE modulation would vary depending on both target speed and distance, and this modulation would be associated with interception timing. In partial support of our hypothesis, we found that the time course of this CSE modulation was influenced by target speed, but not by its starting distance. Notably, after adjusting for movement onset, we observed reduced preparatory suppression and a general increase in excitability for faster-moving targets. This pattern may reflect an enhanced urgency signal that contributes to earlier movement initiation, reduced movement time, and decreased spatial accuracy with increasing target speed. Overall, our results suggest that target visual motion properties modulate corticospinal excitability, playing a crucial role in shaping movement preparation during interception.

Our findings further support that CSE modulation follows a distinct time course of suppression and facilitation during movement preparation. As expected, the timing of both suppression and facilitation in this task was tightly linked with movement onset, with significant MEP suppression relative to baseline observed ~250–300 ms before EMG onset (32, 33, 35, 38, 39, 58). As movement onset approached (~150-50 ms before the target’s arrival), there was a significant increase in CSE in the task-relevant muscle (FDI), consistent with previous studies showing a buildup of excitability immediately before movement execution (41, 46, 59, 60). This facilitatory effect coincides with increased activity measured in M1 during preparation (27, 61). In contrast, the task-irrelevant muscle (ADM) exhibited increasing suppression throughout the preparation phase, suggesting an inhibitory mechanism that optimizes motor output by selectively suppressing unnecessary muscles (34, 37, 45, 58).

Unlike delayed RT tasks, where movement initiation follows an unpredictable cue, the present task allows for active preparation while the target is in motion. Our finding of suppression in this task would therefore go against the impulse control theory of preparatory suppression (33, 34, 40, 62), as withholding a prepared response is not required in our task. Instead, our results support the idea that inhibitory mechanisms within M1 enable effective movement preparation (30, 58). The selective suppression and rapid release of inhibition in task-relevant muscles just before execution may also reflect a surround inhibition or gain modulation mechanism, such that inhibition serves to sculpt corticospinal output underlying precise motor control (32, 38, 57, 63). In the case of interception, this modulation of inhibitory processes likely aids in integrating visual motion information and informs movement timing (64). Altogether, our findings underscore the importance of considering context-specific and dynamic task environments to advance our understanding of the mechanisms underlying preparatory CSE modulation.

Our results agree with previous findings showing a similar ramping of preparatory CSE while timing interceptions under full vision and visual occlusion conditions (47). Their study showed suppression 250–201 ms before movement onset, followed by increasing facilitation within 200–100 ms before movement onset. We observed suppression and facilitation at similar timings (peak suppression ~250 ms and peak facilitation ~50 ms before movement onset) and further showed evidence of some suppression extending as early as 500 ms before movement onset (in the Slow conditions). Interestingly, while we observed sustained and increasing suppression of the task-irrelevant ADM muscle, Marinovic et al. (47) showed no difference in the level of suppression across time points. One possibility for this discrepancy is that, in the present task, participants intercepted on every trial, whereas participants in study by Marinovic et al. (47) were occasionally required to inhibit their prepared response. This additional demand likely engaged differential inhibitory dynamics at the level of M1, perhaps counteracting further suppression of task-irrelevant muscles (46, 65).

Building on this previous work examining CSE while preparing interceptive actions (22, 47), here we measured CSE in scenarios where accurately timing movement onset depends on trial-by-trial estimation of TTC. Our approach allowed us to distinguish between the relative influence of target speed and distance while accounting for the duration of the preparatory phase. Interestingly, though the overall pattern of CSE modulation was similar for both fast- and slow-moving targets, faster targets resulted in less suppression and increased facilitation. Increased CSE facilitation for faster-moving targets may reflect M1 reaching an excitatory threshold sooner, causing an earlier release of motor commands (5, 24, 59). Alternatively, this earlier transition from suppression to facilitation could represent an adjustment to the heightened demands on accuracy when intercepting faster targets (66, 67). In addition, though not significantly different from baseline, we observed some evidence of early preparatory suppression at our earliest tested time point (TMS_500_) only for slow targets, whereas suppression for fast targets relative to baseline only appeared at later time points (TMS_300_ and TMS_250_). This finding is consistent with previous studies showing that shortened preparatory periods (e.g., 350 ms) can eliminate motor-evoked potential (MEP) suppression, while longer preparation times (e.g., 1,400 ms) maintain suppression (68). Thus, it is possible that preparatory suppression is not a universal feature of all movements but rather indicates an adaptive mechanism to optimize response timing. In the present study, when preparing for fast-moving targets, the motor system may prioritize a quicker release of the motor command, enabling timely interception under more urgent conditions (68-70).

When adjusting for movement onset times, the pattern of CSE modulation was comparable between fast and slow targets, but there was an overall heightened excitability for fast targets. This suggests that CSE modulation is more closely associated with the initiation of movement rather than the underlying processes of movement preparation (71). The earlier onset and continued elevation of CSE align with the idea of increased response urgency (72-75) when intercepting fast-moving targets (76, 77), resulting in quicker response initiation (66). The urgency-related CSE modulation observed in the present study may be partially due to the overall shorter duration of preparation when the target is moving faster (68). Nonetheless, we observed some evidence of reduced early suppression and increased late facilitation between fast and slow targets even when the preparation duration was identical, mirroring behavioral differences in movement onset (compare Fast-Far vs. Slow-Close conditions in Figs. 2 and 5). Together, these results suggest that target speed modulates the urgency signal, driving increased excitability for intercepting faster-moving targets (78).

One potential limitation of our study is that as there were only four unique motion conditions, participants may have adopted a strategy based on estimating the target’s initial speed and distance, rather than relying on ongoing visual information. This initial categorization of speed may explain the trend toward earlier suppression for slow target trials. Future studies that vary the predictability of the target’s path or the target’s visual certainty may help determine the extent to which CSE reflects predictive representations of target speed or continuous updating of sensory information (7, 79, 80).

Although we used TMS primarily to examine changes in CSE during interception preparation, we also observed that TMS applied close to target arrival led to delayed movement onset (Fig. 3A), consistent with previous research indicating that TMS can delay RT when applied near the expected response time (41, 43, 81). This delay is typically attributed to the cortical silent period, which temporarily suppresses M1 neuron activity (41, 81). Notably, participants compensated for this delay by shortening their total movement time (compare Figs. 3A and 3B), suggesting that TMS may interfere with motor initiation without affecting online motor control. Interestingly, when TMS was applied 500 ms before target arrival, movement onset occurred earlier compared with blocks without TMS for the three conditions with longer preparation durations. The earlier movement initiation could be attributed to intersensory facilitation, in which the sensory input from the TMS pulse enhances the processing of the imperative stimulus (in this case, integrating visual information about the moving target), thereby speeding up movement onset (30). In contrast, movement onset was not earlier in the Fast-Close condition, likely due to the already constrained movement onset imposed by the short motion duration (750 ms). Further research using dynamic movement execution tasks is needed to clarify the specific mechanisms by which TMS influences motor performance outcomes (82).

### Conclusions

The present study shows that dynamic changes in M1 excitability during preparation play a critical role in using TTC information to trigger accurate interceptive performance, and that alterations in target speed may modulate both the timing and urgency of motor planning and execution. We found that when preparing to intercept a moving target, CSE undergoes a similar transition profile from early suppression to late facilitation, invariant of the speed or distance of the target. However, faster-moving targets led to earlier modulation and heightened excitability, possibly reflecting an increased urgency to respond. The different CSE patterns for fast and slow targets may underline differences in behavioral performance, such that faster-moving targets elicit a faster release of the motor command for earlier movement onset. Furthermore, applying TMS during preparation delayed movement onsets when delivered close to the expected movement onset and sped up movement times. Together, these findings underscore the importance of CSE modulation supporting interceptive actions and highlight how variations in target speed impact motor planning and execution.

## Data Availability

Data will be made available upon reasonable request.
